# Оценка основного обмена у взрослых пациентов с ожирением: возможности и ограничения предсказательных формул

**DOI:** 10.14341/probl13703

**Published:** 2026-05-20

**Authors:** А. А. Аверкина, М. Г. Рафаелян, О. В. Васюкова, Р. М. Гусейнова, П. Л. Окороков, Ю. В. Бурмицкая, В. А. Романова, Д. А. Копытина, Е. П. Атавина-Ермакова, Н. Г. Мокрышева

**Affiliations:** Национальный медицинский исследовательский центр эндокринологии им. академика И.И. Дедова; Endocrinology Research Centre

**Keywords:** основной обмен, непрямая респираторная калориметрия, ожирение, расчетные формулы основного обмена, энергетический обмен, безжировая масса тела, resting energy expenditure, indirect calorimetry, obesity, predictive equations, energy metabolism, fat-free mass

## Abstract

**ОБОСНОВАНИЕ:**

ОБОСНОВАНИЕ. Точная оценка основного обмена (ОО) у пациентов с ожирением и избыточной массой тела является ключевым компонентом персонализированного подбора калорийности питания и физической активности. Несмотря на то, что непрямая респираторная калориметрия (НРК) признана золотым стандартом определения энергетических затрат покоя, в клинической практике преимущественно используются расчетные формулы, точность которых у лиц с ожирением остается ограниченной и вариабельной.

**ЦЕЛЬ:**

ЦЕЛЬ. Сравнить точность оценки ОО с использованием различных предсказательных формул и метода НРК у взрослых пациентов с избыточной массой тела и ожирением.

**МАТЕРИАЛЫ И МЕТОДЫ:**

МАТЕРИАЛЫ И МЕТОДЫ. Проведено открытое одномоментное сравнительное исследование у взрослых пациентов с индексом массы тела (ИМТ) ≥25 кг/м². Основной обмен определяли методом НРК (Cosmed K5) и рассчитывали с использованием восьми распространенных расчетных формул. Композиционный состав тела оценивали методом биоимпедансного анализа (БИА). Для оценки точности применяли среднюю абсолютную ошибку (MAE), среднюю абсолютную процентную ошибку (MAPE), корень средней квадратичной ошибки (RMSE), коэффициент корреляции Пирсона и анализ Бланда-Альтмана.

**РЕЗУЛЬТАТЫ:**

РЕЗУЛЬТАТЫ. В исследование включены 293 пациента (111 мужчин и 182 женщины) с медианой ИМТ 38,3 [32,9; 44,0] кг/м². Медиана ОО, измеренного методом НРК, составила 1964,5 [1570,8; 2370,5] ккал/сут. Все проанализированные расчетные формулы характеризовались значительной индивидуальной погрешностью: средняя абсолютная процентная ошибка превышала 15% более чем у 50% пациентов. Наименьшее среднее смещение относительно измеренного значения отмечалось для формул Roza–Shizgal, WHO (Schofield) и Harris–Benedict, однако даже они демонстрировали широкие границы согласия по данным анализа Бланда–Альтмана. Точность всех формул снижалась с увеличением возраста и степени ожирения и была минимальной у пациентов с ИМТ≥40 и ≥50 кг/м². Формулы, основанные на безжировой массе тела, систематически недооценивали уровень ОО у пациентов с морбидным ожирением.

**ЗАКЛЮЧЕНИЕ:**

ЗАКЛЮЧЕНИЕ. Ни одна из распространенных расчетных формул не обеспечивает высокой индивидуальной точности оценки ОО у взрослых пациентов с ожирением. Даже формулы с минимальным средним смещением характеризуются значительной межиндивидуальной вариабельностью и снижением точности при увеличении возраста и степени ожирения. НРК остается наиболее надежным методом определения энергетических потребностей, особенно у пациентов с морбидным ожирением.

## Abstract (en)

**BACKGROUND:**

BACKGROUND: Accurate assessment of resting energy expenditure (REE) in patients with overweight and obesity is a key component of personalized dietary planning and physical activity prescription. Although indirect calorimetry (IC) is considered the gold standard for measuring resting energy expenditure, predictive equations are predominantly used in clinical practice, despite their limited and variable accuracy in individuals with obesity. AIM: To compare the accuracy of resting energy expenditure assessment using commonly applied predictive equations and indirect calorimetry in adults with overweight and obesity.

**MATERIALS AND METHODS:**

MATERIALS AND METHODS: An open cross-sectional comparative study was conducted in adults with a body mass index (BMI) ≥25 kg/m². Resting energy expenditure (REE) was measured using IC (Cosmed K5) and calculated with eight commonly used predictive equations. Body composition was assessed by bioelectrical impedance analysis InBody770. Accuracy was evaluated using mean absolute error (MAE), mean absolute percentage error (MAPE), root mean square error (RMSE), Pearson correlation coefficient, and Bland–Altman analysis.

**RESULTS:**

RESULTS: The study included 293 patients (111 men and 182 women) with a median (BMI) of 38.3 [32.9; 44.0] kg/m². The median REE measured by IC was 1964.5 [1570.8; 2370.5] kcal/day. All analyzed predictive equations demonstrated substantial individual error, with the mean absolute percentage error exceeding 15% in more than 50% of patients. The smallest mean bias relative to measured values was observed for the Roza–Shizgal, WHO (Schofield), and Harris–Benedict equations; however, even these formulas showed wide limits of agreement in Bland–Altman analysis. The accuracy of all equations declined with increasing age and obesity severity and was lowest in patients with BMI ≥40 and ≥50 kg/m². Equations based on fat-free mass systematically underestimated REE in patients with morbid obesity.

**CONCLUSION:**

CONCLUSION: None of the commonly used predictive equations provides high individual accuracy for estimating REE in adults with obesity. Even equations with minimal mean bias are characterized by pronounced interindividual variability and reduced accuracy with increasing age and obesity severity. IC remains the most reliable method for determining energy requirements, particularly in patients with severe and morbid obesity.

## ОБОСНОВАНИЕ

У взрослых пациентов с ожирением и избыточной массой тела точная оценка величины ОО имеет ключевое значение для разработки персонализированных схем нутритивной поддержки, подбора калорийности рациона и оптимизации физических нагрузок с целью достижения и поддержания клинически значимого снижения массы тела. Метод непрямой респираторной калориметрии (НРК) признан золотым стандартом для определения энергетических затрат в покое [1][2][3][4]. Однако в клинической практике чаще используются расчетные формулы (Харриса–Бенедикта, Миффлина–Сан Жеора, Каннингема и др.), основанные на антропометрических параметрах [4][5][6]. Показатели ОО у лиц с ожирением могут значительно варьировать в зависимости от особенностей состава тела и метаболического статуса, что обусловливает необходимость их валидации в данной популяции. Расчетные формулы часто не учитывают индивидуальные особенности состава тела, такие как вариабельность безжировой массы у лиц с одинаковым ИМТ. Это может приводить к существенным ошибкам в оценке ОО, особенно у лиц с выраженным ожирением. Наряду с традиционными расчетными формулами, для использования которых необходимы стандартные данные антропометрии, в клинической практике также часто используются формулы, учитывающие композиционный состав тела, оцененный с помощью таких приборов, как биоимпедансные анализаторы и, значительно реже — денситометры. Однако такие данные об уровне ОО также являются расчетными, а не измеренными напрямую, и требуют сопоставления с НРК. Сравнительный анализ расчетных формул и данных НРК позволяет оценить точность существующих способов расчета ОО и определить наиболее надежный подход к индивидуальной оценке ОО у пациентов с избыточной массой тела и ожирением, в частности в российской популяции.

## ЦЕЛЬ ИССЛЕДОВАНИЯ

Сравнить точность оценки ОО с использованием различных расчетных формул и метода НРК у взрослых пациентов с ожирением и избыточной массой тела.

## МАТЕРИАЛЫ И МЕТОДЫ

## Место и время проведения исследования

Место проведения. Центр лечения и профилактики метаболических заболеваний и ожирения ГНЦ РФ ФГБУ «НМИЦ эндокринологии им. академика И.И. Дедова», г. Москва.

Время исследования. Исследование проводилось с марта 2024-го по февраль 2025 гг.

## Изучаемые популяции

В исследование включено 293 взрослых пациента (мужчин n=111, женщин n=182) с конституционально-экзогенным ожирением (n=260) и избыточной массой тела (n=33). Всем пациентам проведено амбулаторное комплексное клинико-лабораторное обследование. Клинические характеристики исследуемой выборки пациентов, в том числе в зависимости от пола, представлены в таблице 1.

**Table:** Таблица 1. Характеристики пациентов

	Все	Мужчины (37.9%)	Женщины (62.1%)
Возраст, медиана [ Q1; Q3]	39 [ 29; 47]	38 [ 28; 47]	39 [ 30; 47]
ИМТ, медиана [ Q1; Q3]	38,3 [ 32,9; 44,0]	39,1 [ 33,9; 45,4]	37,7 [ 31,8; 42,3]
Избыточная масса тела	33 (11,3%)	7 (6,3%)	26 (14,3%)
Ожирение 1 степени	62 (21,2%)	24 (21,6%)	38 (20,9%)
Ожирение 2 степени	83 (28,3%)	28 (25,2%)	55 (30,2%)
Ожирение 3 степени (ИМТ 40–49,9 кг/м²)	115 (39,2%)	52 (46,8%)	63 (34,6%)
Морбидное ожирение (ИМТ≥50 кг/м²)	31 (10,6%)	18 (16,2%)	13 (7,1%)

Критерии включения: наличие избыточной массы тела или ожирения (ИМТ≥25 кг/м²), информированное согласие на участие в исследовании.

Критерии исключения: возраст младше 18 лет, острые инфекционные и остро декомпенсированные хронические заболевания, тяжелая почечная недостаточность (рСКФ менее 30 мл/мин/1,73 м²), применение лекарственных средств для лечения ожирения на момент обследования.

## Дизайн исследования

Проведено одноцентровое наблюдательное одномоментное одновыборочное контролируемое несравнительное исследование.

## Методы

Основной обмен (ОО, Resting Energy Expenditure, REE) определяли двумя методами.

**Table:** Таблица 2. Расчетные формулы ОО Примечание. МТ — масса тела, кг; БМТ — безжировая масса тела, кг; Р — рост, см; В – возраст, годы.*ОО в ккал/сут.

Формулы расчета ОО (ккал/сут)	Мужчины	Женщины
Харриса–Бенедикта	66,473 + (13,7516 × МТ) + (5,0033 × Р) – (6,755 × В)	655,0955 + (9,5634 × МТ) + (1,8496 × Р) – (4,6756 × В)
Миффлина–Сан Жеора	(10 × МТ) + (6,25 × Р) – (5 × В) + 5	(10 × МТ) + (6,25 × Р) – (5 × В) – 161
Шофилда (ВОЗ)	18–30 лет: (63 × МТ + 2896)/4,184;30–60 лет: (48 × МТ + 3653)/4,184;старше 60 лет: (49 × МТ + 2459)/4,184	18–30 лет: (62 × МТ + 2036)/4,184;30–60 лет: (34 × МТ + 3538)/4,184;старше 60 лет: (38 × МТ + 2755)/4,184
Кэтча–МакАрдла	370 + (21,6 × БМТ)
Оуэна	879 + (10,2 × МТ)	795 + (7,18 × МТ)
Каннингема	500 + (22 × БМТ)
Дрейера	МТ/0,1015 × В^0,133	МТ/0,1129 × В^0,133
Розы–Шизгала	88,362 + (13,397 × МТ) + (4,799 × Р) – (5,677 × В)	447,593 + (9,247 × МТ) + (3,098 × Р) – (4,330 × В)

Так как в части формул для расчета ОО используется показатель безжировой массы тела (БМТ), в нашем исследовании также проводилась оценка композиционного состава тела с помощью БИА на мультичастотном приборе InBody 770 (InBody Co., Южная Корея).

Расчет ОО выполнен с использованием программного обеспечения Microsoft Excel. Формулы были адаптированы для автоматизированного расчета с учетом введенных данных о поле, возрасте, массе тела и росте пациентов, а также БМТ. Полученные результаты представлены в килокалориях в сутки (ккал/сут).

## Статистический анализ

Статистическая обработка данных проводилась с использованием языка программирования Python 3.10 (библиотек Pandas, NumPy, SciPy, Matplotlib, Seaborn).

Нормальность распределения переменных оценивалась с использованием критерия Шапиро–Уилка. Для сравнения расчетных и измеренных значений ОО использовались: средняя абсолютная ошибка (MAE), средняя абсолютная процентная ошибка (MAPE), корень средней квадратичной ошибки (RMSE), коэффициент корреляции Пирсона (r). Для оценки клинически значимой точности формул рассчитывался процент пациентов, у которых значение MAPE составляло ≤10% и ≤15%.

Сравнение распределений между независимыми группами (например, между мужчинами и женщинами) проводилось с помощью непараметрического критерия Манна–Уитни. Визуализация данных включала построение графиков Бланда–Альтмана с параметрическими границами соглашения (среднее ± 1,96×SD) и линией регрессии.

Групповые различия визуализировались с использованием boxplot-диаграмм, столбчатых графиков, а также сравнительных рейтинговых диаграмм по ключевым метрикам точности. При анализе результатов допускался уровень статистической значимости p<0,05.

## Этическая экспертиза

Протокол исследования одобрен локальным этическим комитетом при ФГБУ «Национальный медицинский исследовательский центр эндокринологии» Минздрава России (выписка из протокола №5 от 13.03.2024 г.).

## РЕЗУЛЬТАТЫ

Уровень измеренного методом НРК ОО составил 1964,5 [ 1570,8; 2370,5] ккал/сутки, дыхательный коэффициент, отражающий соотношение окисления углеводов и липидов, — 0,82 [ 0,78; 0,88] — преимущественно в физиологическом диапазоне. Показатели измеренного ОО в подгруппах по полу и степеням ожирения представлены в таблице 3 и на рисунке 1. В данной выборке отмечался рост уровня ОО в зависимости от ИМТ. При одинаковой степени ожирения мужчины имели более высокие значения медианы ОО, чем женщины, однако статистически значимые отличия отмечались только в группе ИМТ 40–49,9 кг/м². Здесь и далее из подгруппы «ожирение 3 степени» исключены пациенты с ИМТ более 50 кг/м², их данные указаны отдельно.

**Table:** Таблица 3. Уровень основного обмена, %ЖТ и БМТ пациентов в подгруппах по полу и ИМТ

Пол	Группа по ИМТ	ОО, медиана [ IQR]	%ЖТ медиана [ IQR]	БМТ, медиана [ IQR]
Женщины	Избыточная масса тела	1510[ 1280–1826]	41,5[ 39,0–44,4]	44,8[ 41,1–50,0]
Ожирение I	1712[ 1501–1914]	44,9[ 42,2–48,5]	48,2[ 45,9–50,7]
Ожирение II	1847[ 1464–2190]	50,1[ 48,6–51,0]	51,2[ 47,0–56,6]
Ожирение III (40–49,9 кг/м²)	1961[ 1778–2290]	52,2[ 50,9–53,6]	57,2[ 52,4–61,2]
Ожирение III (≥50 кг/м²)	2650[ 2192–3079]	55,2[ 54,3–56,1]	68,9[ 64,5–78,7]
Мужчины	Избыточная масса тела	1657[ 1348–2328]	30,8[ 24,4–32,4]	61,2[ 60,5–65,9]
Ожирение I	1637[ 1459–2306]	35,8[ 33,5–38,1]	68,0[ 62,0–76,3]
Ожирение II	2045[ 1880–2282]	40,0[ 37,1–43,3]	72,1[ 66,9–76,1]
Ожирение III (40–49,9 кг/м²)	2388[ 1999–2671]	45,8[ 41,6–48,8]	76,9[ 71,0–85,0]
Ожирение III (≥50 кг/м²)	2708[ 2434–3344]	52,9[ 50,3–53,7]	86,5[ 81,3–93,1]

**Figure:**
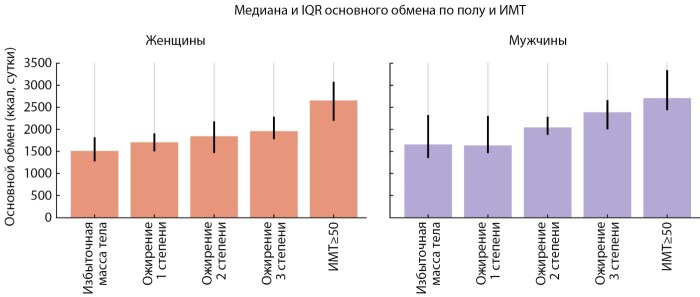
Рисунок 1. Уровень основного обмена пациентов в подгруппах по полу и ИМТ.

Результаты проведенного сравнения точности формул с помощью метода Бланда-Альтмана приведены в таблице 4. Наименьшее смещение (bias) по отношению к измеренному ОО отмечалось у формул Розы–Шизгала (25 ккал), ВОЗ (31 ккал) и Харриса–Бенедикта (HB) (45 ккал), тогда как наибольшая систематическая недооценка была выявлена для формулы Кэтча–МакАрдла (–317 ккал) и Дрейера (–291 ккал). По величине MAE наименьшие значения наблюдались у формул Миффлина–Сан Жеора (372 ккал), ВОЗ (382 ккал), Харриса–Бенедикта (383 ккал) и Розы–Шизгала (384 ккал). Наибольшая MAE зафиксирована у формулы Дрейера (462 ккал) и Кэтча–МакАрдла (450 ккал).

**Table:** Таблица 4. Сравнительные характеристики расчетных формул ОО по отношению к НРК Примечание. Bias — средняя разность между расчетным и измеренным ОО; SD — стандартное отклонение разности; угол наклона — коэффициент линейной регрессии на графике Бланда–Альтмана. MAE — средняя абсолютная ошибка (ккал), MAPE — средняя абсолютная процентная ошибка (%), RMSE — корень из средней квадратичной ошибки (ккал).

Формула	Bias	SD	Угол наклона регрессии	MAE	MAPE, %	RMSE
HB	45,48	485,81	-0,25	383,4	21,1	487,1
MSJ	-88,38	461,95	-0,51	372,1	19,4	469,6
KMA	-316,54	486,2	-0,77	450,1	21,4	579,5
WHO	30,96	486,39	-0,29	382,3	20,9	486,5
Dreyer	-290,84	497,29	-0,26	462,2	23,0	575,4
Owen	-210,86	495,61	-0,5	429,7	21,5	537,8
Cunningham	-186,54	486,2	-0.77	400,0	19,9	520,0
RS	25,02	486,58	-0,26	383,8	20,9	486,4

На рисунке 2 представлен график для формулы HB в качестве примера. Обращает на себя внимание широкий разброс индивидуальных значений измеренного ОО при небольшом с клинической точки зрения среднем смещении (45,48 ккал) расчетных данных по отношению к измеренному методом НРК. Отмечается умеренная зависимость смещения от уровня обмена для формулы HB, для остальных формул зависимость более выраженная. (см. угол наклона регрессии в таблице 4).

**Figure:**
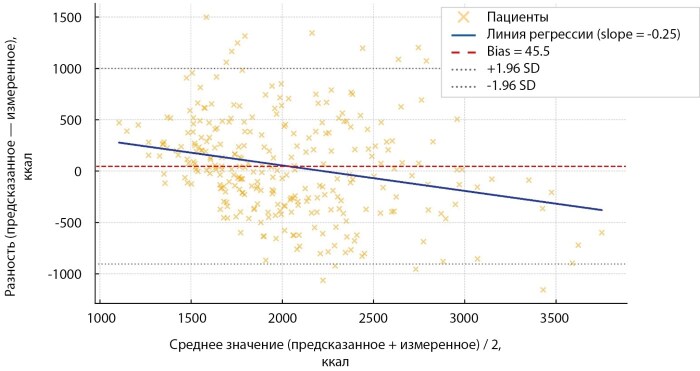
Рисунок 2. График Бланда–Альтмана с линией регрессии (формула HB). График демонстрирует расхождение между расчетным и измеренным ОО по формуле Harris–Benedict. Синяя линия отражает регрессионный тренд, свидетельствующий о систематической ошибке при высоких значениях ОО.

С целью дальнейшей оценки точности расчетных формул на индивидуальном уровне были рассчитаны MAE, MAPE, RMSE и коэффициент корреляции Пирсона r для всей выборки (табл. 4), а также отдельно по половозрастным группам и по подгруппам степеней ожирения.

На рисунке 3А наглядно представлено, что во всех возрастных группах у женщин все формулы предсказывают уровень ОО с MAPE более 15%, при этом с возрастом точность всех формул снижается. Аналогичные данные были получены среди мужчин (рис. 3Б).

**Figure:**
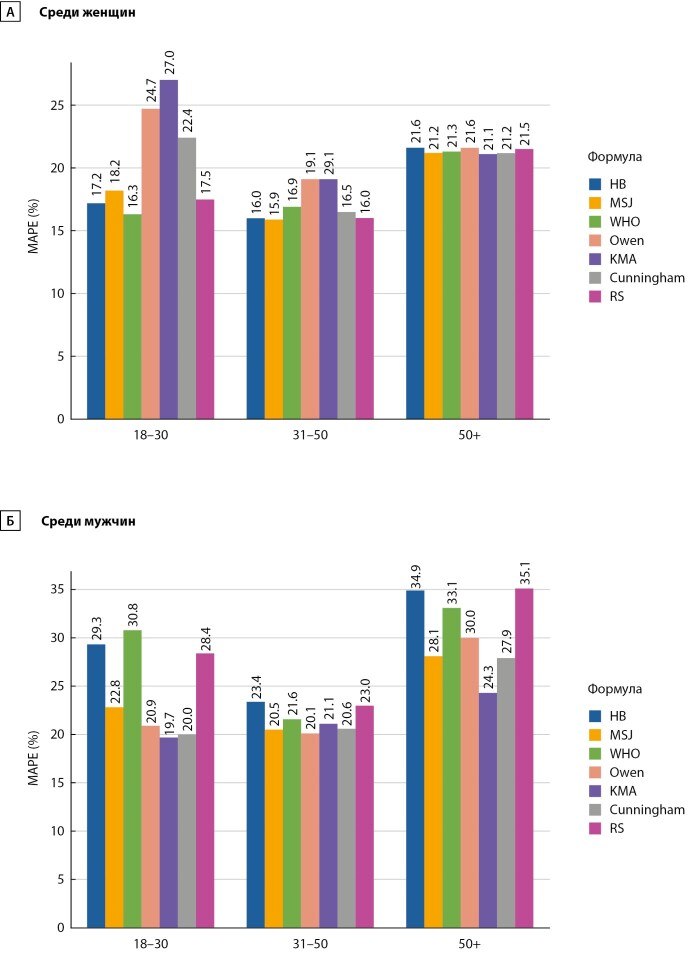
Рисунок 3. Средняя абсолютная процентная ошибка (MAPE) в возрастных подгруппах для различных расчетных формул ОО. По оси х — возрастные подгруппы 18–30 лет, 31–50 лет и старше 50 лет соответственно. В старшей возрастной группе — наибольшая ошибка — более 20% для всех анализируемых формул.

С целью сравнения индивидуальной точности формул с учетом пола и возраста проанализировано, какие формулы дают ошибку менее 10% и менее 15% по сравнению с НРК реже остальных. На рисунке 4 представлено процентное количество пациентов, для которых каждая из формул оказалась относительно точной с клинической точки зрения среди женщин и мужчин (а и б соответственно). Видно, что для большинства подгрупп формулы оказывались точны для 50% пациентов и менее. В таблицах 5 и 6 вынесены наилучшие формулы по данному показателю. Так, в группе молодых женщин от 18 до 29 лет самой точной оказалась формула ВОЗ, предсказав уровень ОО с погрешностью в пределах 10% в 40,0% случаев, и в пределах 15% в 57,8% случаев. Интересно также, что наилучшей точностью в группе молодых мужчин обладают формулы Кэтча-МакАрдла и Каннингема, тогда как в общей выборке они характеризуются наибольшими значениями всех видов ошибок и наибольшим углом наклона линии регрессии.

**Figure:**
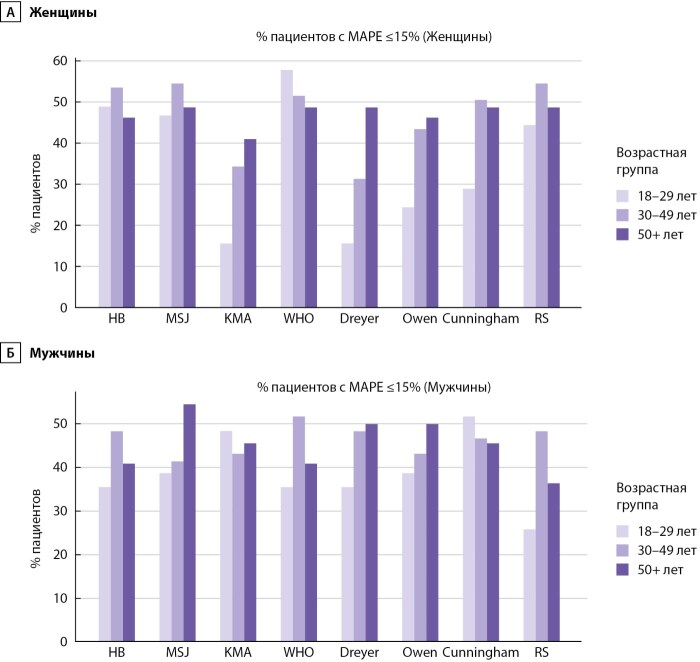
Рисунок 4. Доля пациентов с MAPE ≤ 15% по полу и возрастным группам.

**Table:** Таблица 5. Формулы-фавориты по возрастно-половым группам на основе процента пациентов с MAPE ≤15% В каждой группе выбрана формула, обеспечившая наибольший процент пациентов, у которых MAPE не превышала 15%.

Пол	Возрастная группа	Формула	% с MAPE ≤ 15%
Женщины	18–29 лет	WHO	57.,8
30–49 лет	MSJ	54,5
50+ лет	MSJ	48,7
Мужчины	18–29 лет	Cunningham	51,6
30–49 лет	WHO	51,7
50+ лет	MSJ	54,5

**Table:** Таблица 6. Формулы-фавориты по возрастно-половым группам на основе процента пациентов с MAPE ≤10% В каждой группе выбрана формула, обеспечившая наибольший процент пациентов, у которых MAPE не превышала 10%.

Пол	Возрастная группа	Формула	% с MAPE ≤ 10%
Женщины	18–29 лет	WHO	40,0
30–49 лет	HB	39,4
50+ лет	HB	35,9
Мужчины	18–29 лет	KMA	38,7
30–49 лет	RS	39,7
50+ лет	WHO	36,4

Исключение из анализа пациентов с ИМТ более 35 кг/м² не привело к значительному улучшению точности расчетных формул. Так, для формул HB и RS различия в ошибках между подгруппой с ИМТ<35 кг/м² и всей выборкой статистически незначимы (p>0,05). Только для формулы KMA MAE статистически значимо ниже в группе с ИМТ<35 кг/м² (p=0,005), что говорит о ее меньшей применимости при выраженном ожирении.

## ОБСУЖДЕНИЕ

Результаты настоящего исследования демонстрируют, что при индивидуальном применении у пациентов с избыточным весом и ожирением среднего возраста, формулы для расчета ОО имеют ограниченную точность, особенно у пациентов с морбидным ожирением. Наши данные свидетельствуют о высокой энергетической потребности у лиц с морбидным ожирением: уровень ОО у пациентов с ИМТ>50 кг/м² составил 2650 [ 2192–3079] у женщин и 2708 [ 2434–3344] ккал/сутки у мужчин. Аналогичные данные приводятся в работе Wu и соавт., где подчеркивается, что пациенты с ожирением III степени демонстрируют значительно более высокие показатели ОО, чем можно ожидать при расчете по классическим формулам [16]. Авторами метаанализа 2024 г. также отмечено, что с наименьшей погрешностью ОО можно рассчитать для пациентов с избыточной массой тела [4].

Несмотря на отсутствие выраженного систематического смещения между средними значениями измеренного и расчетного ОО (например, 45,5 ккал/сут по формуле HB), анализ по методу Бланда–Альтмана, а также оценка MAPE выявили значительный разброс индивидуальных различий. Это свидетельствует о недостаточной точности большинства формул при клиническом применении у конкретного пациента. Подобные выводы были сделаны и в других работах: предиктивные уравнения, включая формулы HB, Мифлина–Сан-Жеора и ВОЗ, не обеспечивают высокой точности оценки ОО у женщин с ожирением, особенно при морбидном [5][17].

Анализ показателей MAE, MAPE и RMSE продемонстрировал, что ни одна из оценочных формул не обеспечивает приемлемой точности (MAE<200 ккал, MAPE≤15%) на индивидуальном уровне. Особенно низкая точность наблюдалась в нашем исследовании у женщин и в старших возрастных группах, что может быть связано с возрастными изменениями в составе тела или гормональными нарушениями, а также с составом выборок, на которых разрабатывались исходно анализируемые формулы. Схожую низкую индивидуальную точность формул Харриса–Бенедикта, Мюллера и Лейзера отметили в исследовании Rodrigues et al. у пациенток с синдромом поликистозных яичников, имеющими избыточную массу тела или ожирение [6].

Дополнительный анализ показал, что только для 50% пациентов расчетные значения ОО отличались от измеренных менее чем на 10–15%. Наибольшая точность наблюдалась при использовании формулы ВОЗ у женщин молодого возраста (18–29 лет), а у мужчин — формул KMA и Каннингема. Однако для пациентов с ИМТ>35 кг/м² точность формулы KMA была существенно ниже. Более того, выявлен тренд к систематическому занижению ОО при увеличении истинных значений, что делает ее использование у пациентов с выраженным ожирением небезопасным с точки зрения риска гипокалорийного питания. При этом для части пациентов формула KMA завышала значение ОО — возможно, это связано с тем, что с помощью БИА при морбидном ожирении затруднительна диагностика саркопенического ожирения — снижения мышечной массы при одновременном увеличении жировой ткани. В таких случаях применение формул, основанных на массе тела или жировой массе, приводит к искажению результатов, так как мышечная масса является главным детерминантом уровня ОО. Потеря мышечной ткани усиливает риск гипердиагностики энергетических потребностей, что особенно критично при диетотерапии и может способствовать развитию нутритивной недостаточности.

Исключение из анализа пациентов с ИМТ>35 кг/м² привело к статистически значимому улучшению точности для формулы KMA, основанной на измерении безжировой массы. Это может быть объяснено с нескольких позиций. Во-первых, при выраженном ожирении увеличивается масса не только жировой ткани, но и некоторых органов с высокой метаболической активностью — печени, почек, сердца, что вносит вклад в повышение уровня основного обмена [18]. Во-вторых, жировая ткань, особенно при гипертрофии и хроническом воспалении, возможно, становится более метаболически активной, вырабатывая широкий спектр адипокинов (включая лептин, ИЛ-6, TNF-α), которые модулируют обмен веществ и усиливают симпатическую активацию [19][20]. Таким образом, полученные нами данные подтверждают, что с нарастанием степени ожирения вклад массы тела в предсказание уровня ОО увеличивается, тогда как значимость безжировой массы ослабевает, несмотря на ее физиологическую роль как основного метаболически активного компонента.

Таким образом, полученные данные указывают на ограниченную применимость стандартных предиктивных уравнений для оценки ОО у пациентов с ожирением, особенно в подгруппах с ИМТ>35 кг/м² и у женщин старших возрастных категорий.

Прямое измерение ОО с использованием НРК остается наилучшим методом оценки ОО, особенно для пациентов с морбидным ожирением. Когда НРК недоступна, допустимо использование расчетных формул, однако в связи с высокой индивидуальной вариабельностью уровня ОО в любом случае необходима динамическая клиническая переоценка эффективности назначенной энергетической поддержки — по самочувствию пациента, динамике массы тела и изменению состава тела, прежде всего безжировой массы.

## Ограничения исследования

К ограничениям исследования можно отнести то, что набор участников исследования проводился только в федеральном научном центре среди пациентов специализированного подразделения, которые прежде неоднократно предпринимали попытки снижения массы тела различными способами.

## ЗАКЛЮЧЕНИЕ

Результаты настоящего исследования демонстрируют, что ни одна из восьми распространенных формул расчета ОО не обеспечила высокой индивидуальной точности у пациентов с ожирением. В большинстве подгрупп — как по полу, так и по возрасту и степени ожирения, — доля пациентов, для которых ошибка прогноза (MAPE) не превышала 10%, составляла менее 50%, а в ряде случаев — менее 30%. Подобные данные согласуются с результатами опубликованных ранее работ, указывающих на значительную вариабельность энергетических потребностей у лиц с ожирением [4][5][21][22]. В нашем ранее выполненном исследовании [22] проводилось сравнение точности расчетных формул и метода НРК у детей с простым ожирением. Было показано, что, несмотря на относительно приемлемую точность некоторых уравнений в группах с избыточной массой тела, при выраженном ожирении систематическая ошибка расчетных формул значительно возрастает. Особенно низкая точность была отмечена у пациентов с более высоким ИМТ, что подтверждает необходимость использования методов прямого измерения ОО в клинической практике.

Наиболее точными формулами для взрослых пациентов в нашей выборке оказались Mifflin–St Jeor (MSJ) [9], WHO (Schofield) [11] и Roza–Shizgal (RS) [15] (и схожая с ней формула Харриса–Бенедикта). Однако даже они демонстрировали значительное индивидуальное расхождение с измеренными значениями ОО, особенно у мужчин молодого возраста до 30 лет (для которых предпочтительнее использование формул с учетом БЖТ KMA и Каннингема) и пациентов с морбидным ожирением (ИМТ≥40 кг/м²). Высокие значения RMSE и MAE указывают на то, что применение расчетных формул в этих группах может приводить к клинически значимым ошибкам. В исследовании, в котором проводилась оценка точности формул у пожилых пациентов (60–75 лет) с выраженным ожирением в Италии, также отмечалась более высокая точность у расчетных формул на основе антропометрических показателей, а не компонентов состава тела [23].

Полученные данные подчеркивают ограничения расчетных методов и подтверждают высокую практическую значимость НРК как золотого стандарта метода оценки метаболических потребностей у пациентов с ожирением [24][25]. Особенно актуально применение НРК в ситуациях, требующих индивидуализации питания — в послеоперационном периоде, в отделениях интенсивной терапии, при ожирении 3 степени (особенно с ИМТ более 50 кг/м²) [25], у пациентов с подозрением на саркопеническое ожирение [26].

## ДОПОЛНИТЕЛЬНАЯ ИНФОРМАЦИЯ

Источники финансирования. Работа выполнена в рамках государственного задания «Механизмы развития эффекта «плато» после снижения массы тела и рецидива ожирения у детей и взрослых: адаптивный термогенез, миокиновый профиль, пищевое поведение, метаболические, нутритивные и провоспалительные маркеры», регистрационный номер 1023022400038-1

Конфликт интересов. Авторы декларируют отсутствие явных и потенциальных конфликтов интересов, связанных с публикацией настоящей статьи.

Участие авторов. Все авторы одобрили финальную версию статьи перед публикацией, выразили согласие нести ответственность за все аспекты работы, подразумевающую надлежащее изучение и решение вопросов, связанных с точностью или добросовестностью любой части работы.

Благодарности. Авторы выражают искреннюю благодарность пациентам, принявшим участие в проведении исследования.
